# How we do it: Ultrasound-guided scuba technique for evacuation of intracerebral hematoma

**DOI:** 10.1007/s00701-025-06539-y

**Published:** 2025-05-02

**Authors:** Alejandra Mosteiro, Alberto Di Somma, Luis Reyes, Ana Rodríguez-Hernández, Ramon Torné

**Affiliations:** 1 Department of Neurosurgery, Hospital Clínic de Barcelona, Barcelona, Spain; 2 Faculty of Medicine, University of Barcelona, Barcelona, Spain; 3 August Pi I Sunyer Biomedical Research Institute (IDIBAPS), Barcelona, Spain; 4 Department of Neurosurgery, Hospital Germans Trias I Pujol, Badalona, Barcelona, Spain

**Keywords:** Intracerebral hematoma, Minimally invasive, Endoscopic, Stereotactic, Ultrasound

## Abstract

**Background:**

Minimally invasive treatment of intracerebral hematomas is gaining importance following recent trials. Clot evacuation must minimise collateral damage while assuring optimal blood-volume reduction. Technical refinements call for systematic yet dynamic procedures, where ultrasound is an asset.

**Method:**

Description of the “Scuba” procedure and workflow: stereotactic canula insertion, clot evacuation with aspiration-debriding system under endoscopic view. Step-by-step ultrasound findings detailed.

**Conclusion:**

ICH endoscopic evacuation may become a workhorse for cerebrovascular surgeons. Ultrasound provides real-time feedback on hematoma evacuation, becoming a reliable dynamic element in intraoperative decision-making. Yet, US requires a different operative setup and adds on to the learning curve.

**Supplementary Information:**

The online version contains supplementary material available at 10.1007/s00701-025-06539-y.

## Relevant surgical anatomy

The perihematomal area is a strategic interface for potentially reversible deleterious effects known as secondary brain damage. This area, now considered homologous to penumbra in ischemic stroke, is the salvageable target of early MIS evacuation [9].

From STICH trials we learned collateral damage to healthy brain counteracts potential benefits of clot removal [4]. Therefore, building upon knowledge of stereotactic neurosurgery, we must ensure a trajectory as direct and safe as possible. After MISTIE trial we understood a threshold of evacuation is needed for it to be effective, presumably 70% of initial volume [2]. The ENRICH trial demonstrated us that active aspiration under direct endoscopic view provides better results than placement of passive catheters [7]. Upcoming trials like DIST will shed light on unmet questions such as optimal time window or benefits in basal ganglia hematomas [8].

State-of-art ICH evacuation is reshaping and broadening the technical know-how of cerebrovascular surgeons. Remarkably as the procedure is to be performed in an emergency setting. Comprehension of different surgical techniques available along with intraoperative imaging tools is essential to custom surgeon’s practice [5]. The parafascicular procedure through a tubular retractor is described by the Basel group in this journal [6].

We describe the stereotactic endoscopic underwater evacuation, through a 19 F sheath and an aspiration-debriding system. Intraoperatively, our decision-making largely relies on ultrasound (US), as here detailed, while final control is done with intraoperative CT.

## Technique

### Trajectory

All patients undergo a preoperative thin-slice (0.5 mm) basal CT and CTA to rule out subjacent lesions and for trajectory planning. Images are transferred to BrainLab station, and trajectory is planned; meanwhile, the patient is being transferred to the operating room and anesthetised.

Principles for trajectory planning: 1) Follow the longitudinal axis of the haematoma; 2) endpoint is set 1 cm before the distal margin of the clot; 3) intracerebral course should be as short as possible and avoid sulcus, ventricles and vessels (CTA); for basal ganglia lesions, a frontal entry point is usually chosen [10].

The trajectory length should be noted as a back-up safety reference intraoperatively (not to surpass it with the scope).

### Set up

**Fig. 1:**
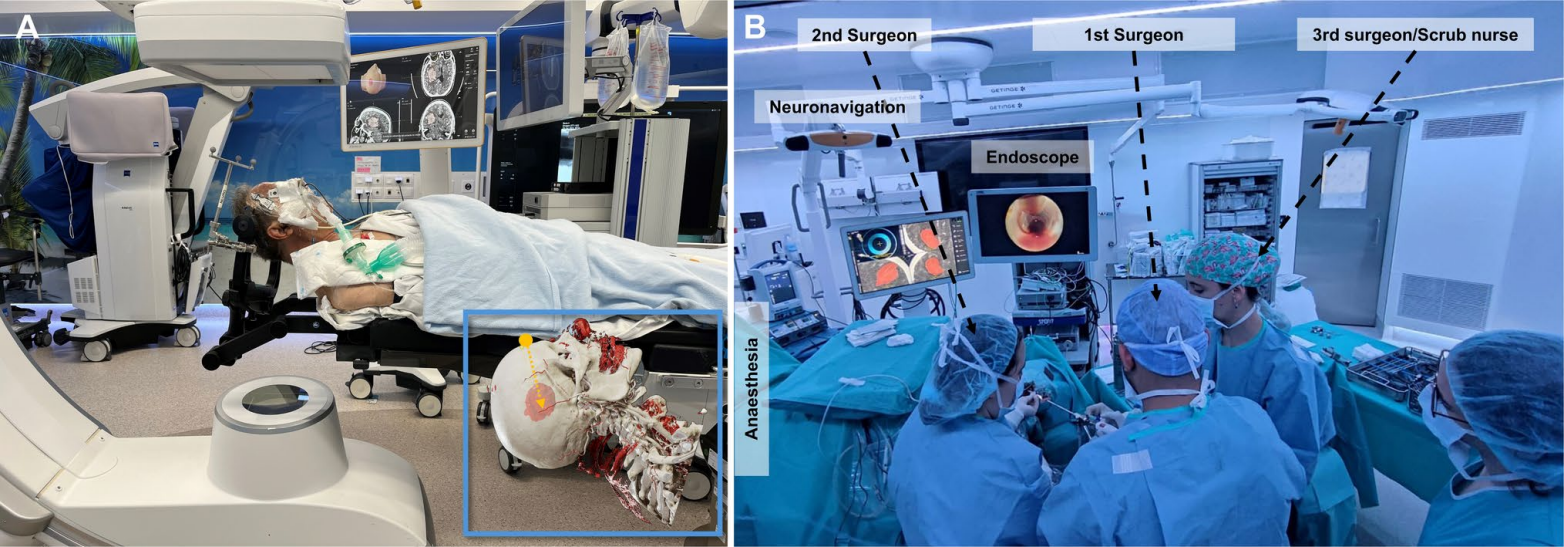
Room set up. The images correspond to the same case shown in Fig. 1. **A** The patient is positioned so that the planned entry is at the highest point (see superimposed 3D CTA reconstruction with trajectory marked in orange). The head is clamped. The neuronavigation is set up guaranteeing that the camera will recognise the passive reference, and the tracker attached to the endoscope working port (canula) at all times.** B** The first surgeon should face the endoscope tower, while the second surgeon should face the neuronavigation screen

Room set should guarantee neuronavigation is always working, and both the passive frame and the tracker pointer attached to the canula *are visible at all times*. Ergonomics should be so that endoscope screen faces the primary surgeon and neuronavigation screen faces the secondary surgeon (Fig. 1).

Patient should be positioned so that the entry is at the highest point, for manoeuvrability and visualization, as otherwise blood dropping keeps opacifying the endoscope lens. Head is clamped, and neuronavigation registration done.

The neuronavigation tracker is attached to the canula (working port of the endoscope) and registered. The endoscope is attached to a water-pump and either connected to an ICP measurement line, or a side connection of the scope is left opened to allow retrograde flow from the cavity while irrigating. The evacuation system (Artemis, Penumbra Inc.) is checked.

### Craniotomy and hematoma measurements

**Fig. 2:**
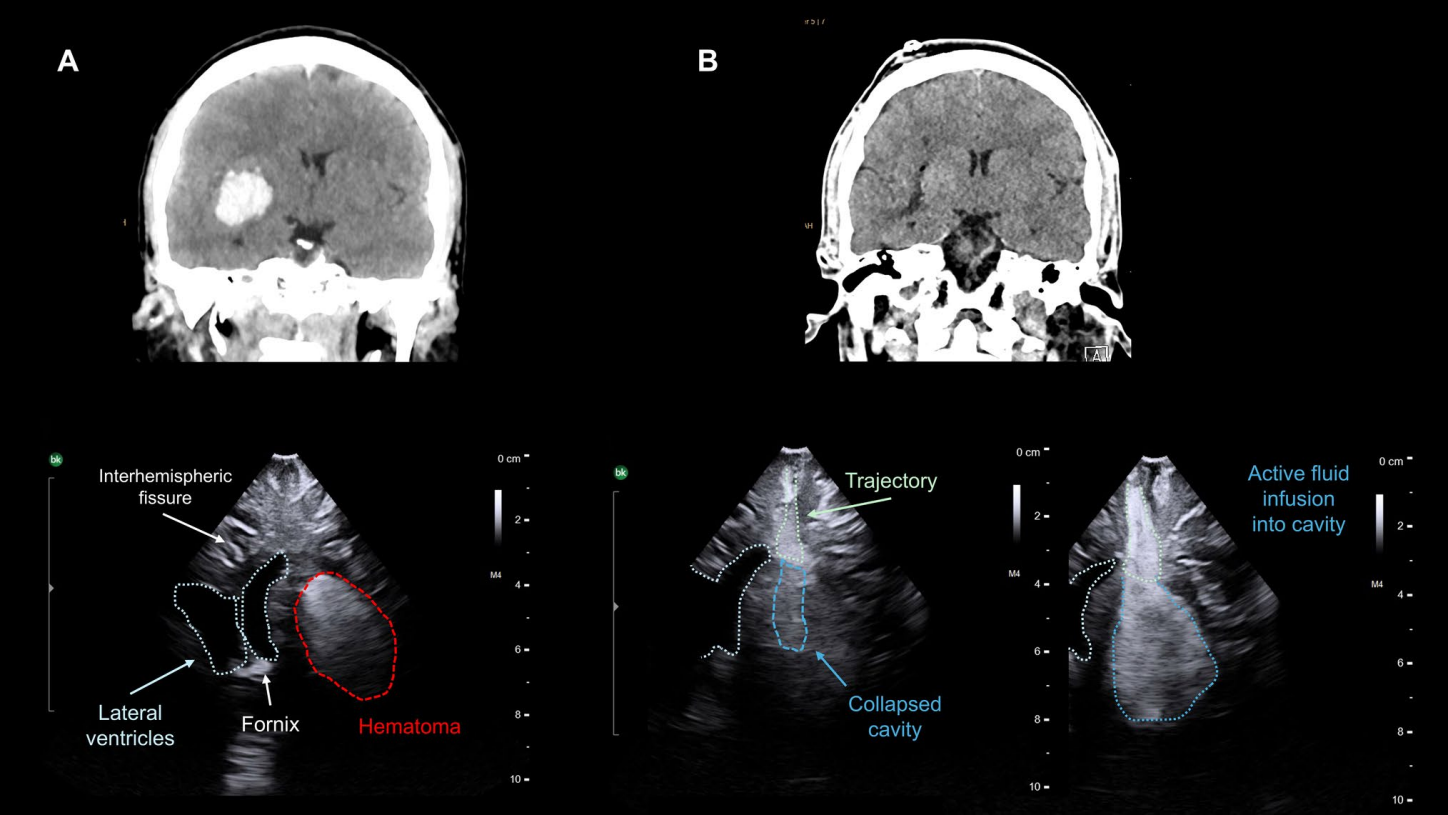
Complete evacuation of the hematoma as seen with intraoperative US. **A** Above, the basal CT scan showing a 20 cm3 right basal ganglia hematoma. Below, the corresponding US image obtained through a mini-craniotomy before opening the dura. Note that the lateral ventricles can be used for orientation in order to identify the hematoma. The hematoma appears as an hyperechogenic oval structure; the deeper portion may be hypoechogenic due to an acoustic shadow effect. **B** Above, postoperative CT showing complete hematoma evacuation after one pass of aspiration. Below, images obtained with US after the first pass of aspiration. On the left, the cavity is collapsed, and the acoustic signal is similar to the surrounding parenchyma. On the right, the cavity is being filled with saline solution infused with a syringe through the trajectory path. Note the hyperechogenic and homogeneous appearance of the dilated saline-filled cavity

**Fig. 3:**
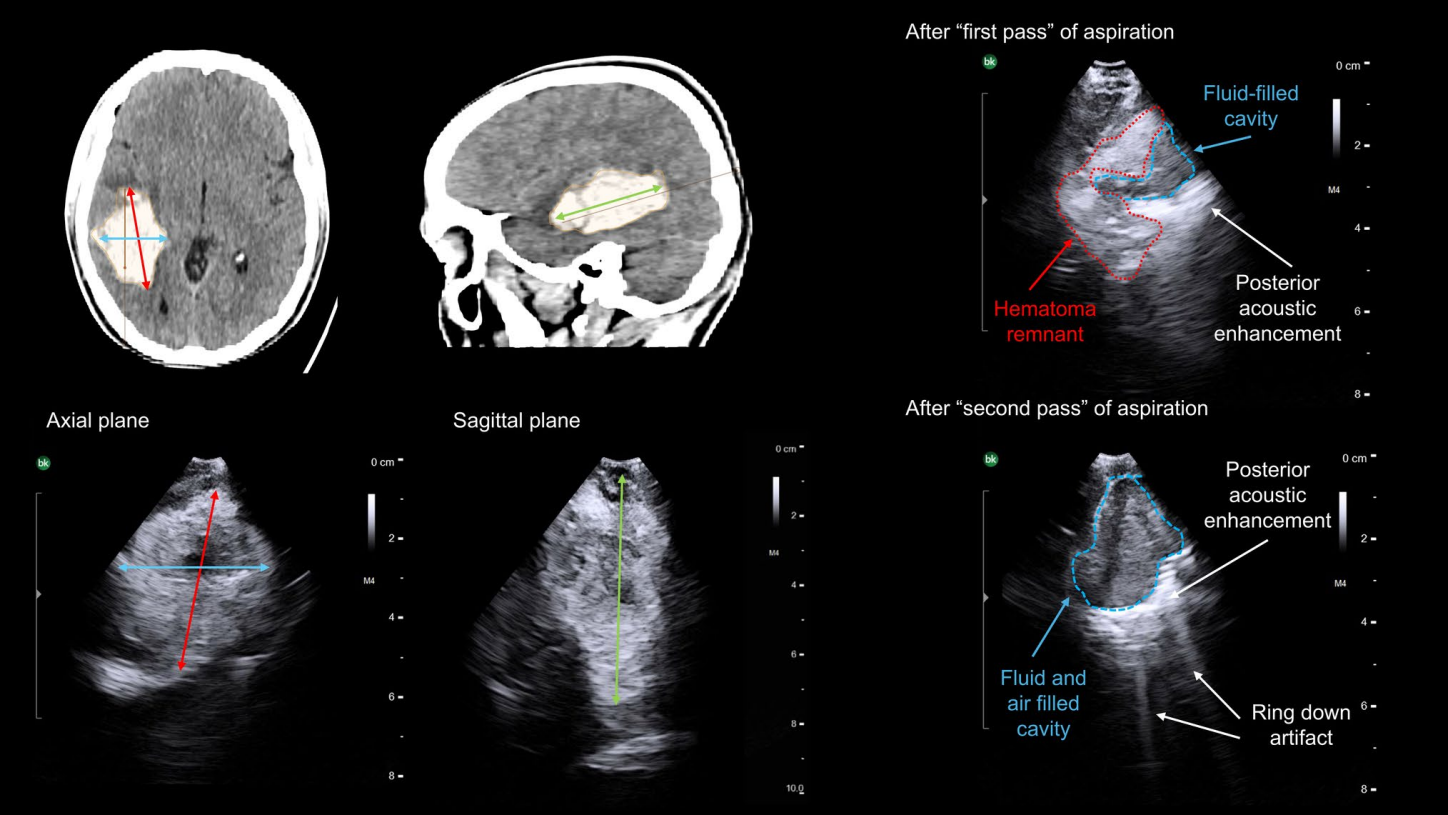
Hematoma remnant after a first pass of aspiration as detected with intraoperative US. The basal CT scan (axial and sagittal) of a patient with a 47 cm3 right temporal hematoma is shown on the upper left. The initial volume is calculated intraoperatively with US as shown in the lower left of the panel (red, blue and green representing the three dimensions of the hematoma). On the upper right, the US imagen after a first pass of aspiration is shown. Note that the cavity appears hypoechogenic, with a posterior hyperechogenic acoustic enhancement. The hematoma remnant can be delineated in the superolateral margin of the cavity, with an acoustic intensity like that at baseline. After a second pass of aspiration (lower right corner of the panel), the cavity appear hypoechoic, no clot remnants are seen. Note the posterior acoustic enhancement due to the presence of liquid (saline) in the cavity. Also note the ring-down artifact (“irregular linear hyperechogenic lines”) provoked by the presence of air within the cavity

Patient is given preoperative antibiotics and seizure prophylaxis. Entry point is localised with navigation and a mini-craniotomy or amplified burr hole is drilled (20 × 20 mm), enough to allocate the ultrasound (US) probe (Burr-Hole- 8863, BK-Medical). Depth and penetrance of US are adjusted to size and location of the hematoma. Constant anatomical landmarks, like ventricles or falx, are useful for orientation (Fig. 2). The probe can be tilted at the craniotomy margins to identify all the hematoma limits. Clot is measured in the three planes with US **(**Fig. 3) and initial volume calculated with ABC/2 rule; coronal and sagittal projections attained by modifying probe direction. Dura is opened in a cruciform fashion only at the entry point for the endoscope.

### Neuronavigation-guided endoscopic evacuation

The hematoma is virtually divided into quadrants for the purposes of this technique. The 19 F canula is introduced, under neuronavigation, into one of them. The endoscope (LOTTA®, Karl-Storz) is introduced, the clot visualised and evacuated by aspiration, debriding, and saline irrigation. The initial phase usually requires more aspiration (liquid clot type) and/or debriding (dense clot type); while the second phase, once parenchyma is reached, requires saline flush to detect and control bleeding. As parenchyma is seen, the canula and endoscope are delicately moved to the next quadrant and the same sequence applies. Evacuation continues in this fashion from distal to proximal within the hematoma and finishes when cavity collapses as the canula reaches the proximal margin of the clot (Video).

### Decision-making with US guidance

After completing the “first pass” of aspiration, US is used to identify any remaining hematoma (Fig. 2). If evacuation has been significant, the subdural space spontaneously opens as a sign of good prognosis. Presence of subdural air requires filling with saline before US probe is placed.

Location of hematoma remnant is noted in relation to previously established quadrants. Its dimensions are measured and if evacuation is considered insufficient (< 70% of initial volume), a second (even third pass) is deemed and directed into the suspected quadrant. After satisfactory evacuation, based on US, an intraoperative high-resolution 3D X-ray scan is done (Azurion SmartCT, Philips) to confirm evacuation and rule out complications.

**Fig. 4:**
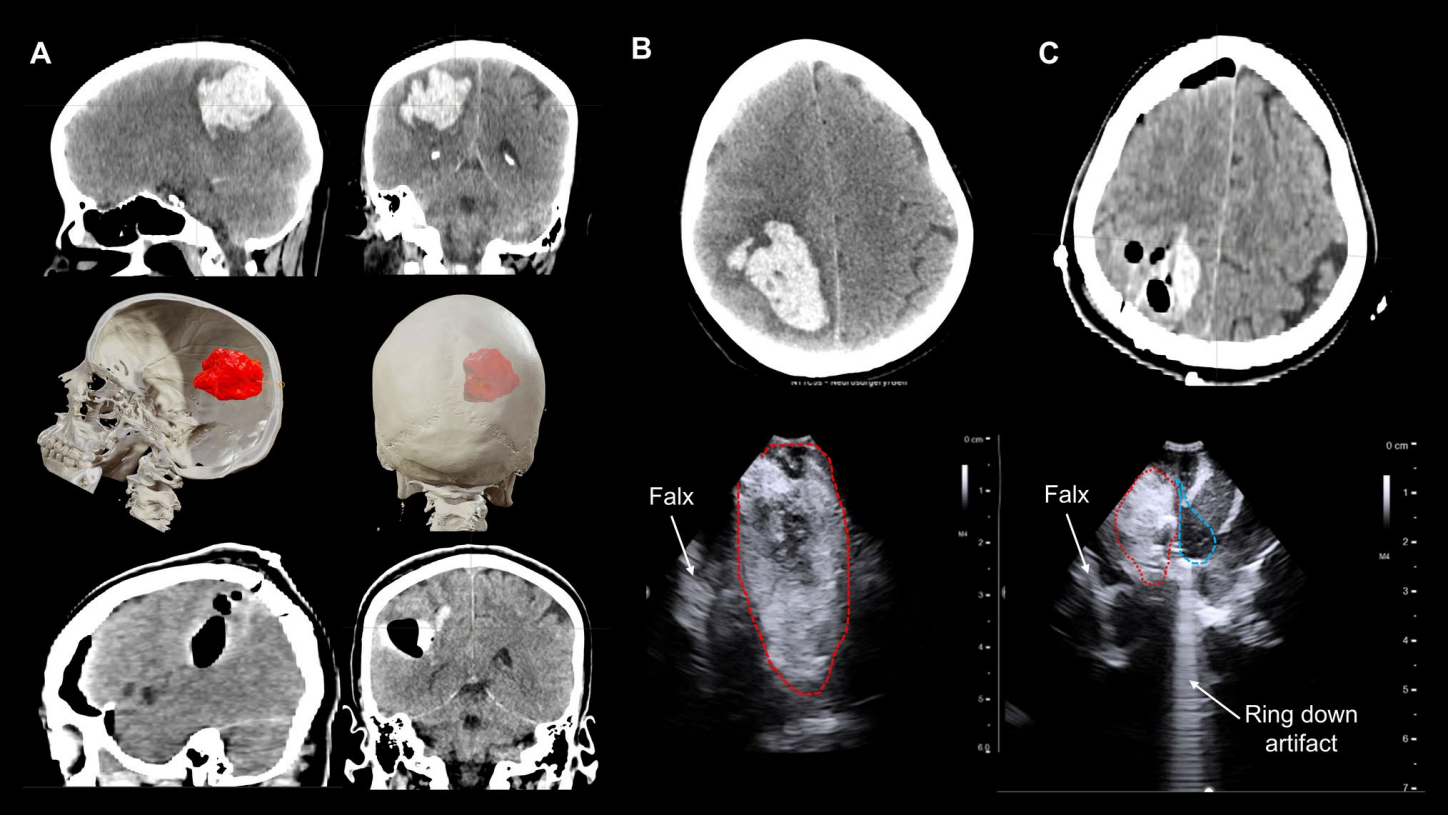
Correlation between intraoperative US findings and postoperative CT scan. **A** On the first row, basal sagittal and coronal CT projections showing a right parietal hematoma (45 cm3). The middle row shows the 3D reconstructions of the CT scan and the planned trajectory, with the entry point near lambda; the patient was positioned in a lateral decubitus. The bottom row shows the postoperative CT scan. **B** Axial projection of the CT scan of the same patient; see below the intraoperative US, where the falx is used as a reference for orientation. **C** Postoperative axial CT scan showing a clot remnant in the medial aspect of the cavity. Note below how the intraoperative US reproduces these findings, with a hematoma remnant seen in the medial part of the cavity, close to the falx. Also note the ring-down artifact (“irregular linear hyperechogenic lines”) provoked by the presence of air within the cavity

US merits a learning curve as a useful tool particularly in the emergency setting, able to replicate CT findings quite accurately (Fig. 4). Several acoustic artifacts might indicate optimal clot evacuation. The presence of saline in the cavity creates an “acoustic enhancement” sign. Further filling of the cavity with saline generates an hyperechogenic homogeneous expansion of the cavity. While the presence of air creates a “dirty shadow"or “ring down” artifact, as sound waves traverse the bubble-fluid collection (Figs. 2, 3 and 4).

## Indications

In our centre, indications are primary supratentorial ICH (negative CTA), ≥ 20 mL, mild-moderate neurological impairment (GCS 8–14 and/or NIHSS ≥ 6), previously independent (mRS0 - 2). Exclusion criteria include coma (GCS < 8) or signs of irreversible brainstem damage, secondary lesions, infratentorial or primary thalamic, high-risk of thrombosis, irreversible coagulopathies, refractory hypertension.

Based on our experience and preliminary evidence of a time-dependent effect, we have moved from a policy of stability-confirmation (control CT 6 h after onset) towards early evacuation (within the first 8 h) under the concept of “haemorrhagic code stroke” [1].

## Limitations

Use of intraoperative US might require a bigger burr hole or mini-craniotomy to accommodate the probe. To minimise invasiveness and brain shift effect, dura is only opened in the point of endoscope insertion.

## How to avoid complications

Adequate candidate selection, efforts driven to detect subjacent lesions, and preoperative management of coagulation. Trajectory planning should consider surgical manoeuvrability, avoiding extreme angulations. Intraoperatively, constant visualisation of neuronavigation screen by one surgeon is paramount; limitations inherent to navigation must be observed, particularly brain shift. In active bleeding, taking the endoscope out is unadvisable; profuse saline irrigation will, in our experience, stop the bleeding. Otherwise, if arterial bleeding is discovered, endoscopic bipolar forceps could be used.

## Specific information for the patient

MIS evacuation of ICH is a change in paradigm. Although it seems to improve mortality and functional outcomes, this is yet to be consolidated, particularly in basal ganglia haemorrhages [8]. The objectives of surgery must be realistically stated.

Although the procedure appears safer than conventional craniotomy evacuation, potential complications should be explained, especially the need for intraoperative craniotomy conversion or reintervention due to active rebleeding.

## Supplementary Information

Below is the link to the electronic supplementary material.

Supplementary file1 (MP4 297607 KB)

## Data Availability

No datasets were generated or analysed during the current study.

## Abbreviations

*CT*: Computed Tomography
*CTA*: Computed Tomography Angiography
*DSA*: Digital Subtraction Angiogram
*GCS*: Glasgow Coma Scale
*ICH*: Intracerebral Haemorrhage
*MIS*: Minimally Invasive Surgery
*NIHSS*: National Institutes of Health Stroke Scale
*US*: UltraSound
